# The psychological basis of reductions in food desire during satiety

**DOI:** 10.1098/rsos.221404

**Published:** 2023-05-24

**Authors:** Richard J. Stevenson, Heather M. Francis, Fiona Wylie, Alannah Hughes

**Affiliations:** Department of Psychology, Macquarie University, Sydney, New South Wales 2109, Australia

**Keywords:** desire, satiety, diet, learning, associative, inhibition

## Abstract

Satiety—the reduced desire to eat, drink or have sex in their respective aftermath—is particularly important for feeding, where it assists energy balance. During satiety, the anticipated pleasure of eating is far less than the actual pleasure of eating. Here we examine two accounts of this effect: (i) satiety signals inhibit retrieval of pleasant food memories that form desirable images, allowing unpleasant memories into mind; (ii) feelings of fullness reflect what eating would be like now, negating the need for imagery. To test these accounts, participants undertook two tasks pre- and post-lunch: (i) judging desire for palatable foods either with or without imagery impairing manipulations; (ii) explicitly recollecting food memories. Impairing imagery reduced desire equally, when hungry and sated. Food-memory recollections became more negative/less positive when sated, with this correlating with changes in desire. These findings support the first account and suggest imagery is used when hungry and when sated to simulate eating, and that the content of these memory-based simulations changes with state. The nature of this process and its implications for satiety more generally are discussed.

## 1. Introduction

Humans and animals are less likely to eat immediately after a meal, less likely to drink immediately after quenching a thirst, less likely to have sex immediately following intercourse, and less likely to use a particular narcotic immediately after using it (e.g. [1–4]). All are examples of satiety, where desire for more of a particular thing is dulled in its immediate aftermath. In this manuscript, we examine some of the psychological processes that occur during satiety, and we focus in particular on those involving food. This example is highly consequential, as eating when sated is a potentially important contributor to excess weight gain (e.g. [5]), with obesity remaining a significant global health problem (e.g. [6]).

Desire, or as it is more properly referred to—wanting—normally becomes dulled after a meal (e.g. [7]). This is functionally useful, as if a food is encountered when sated, it is unlikely to be tasted if it seems unappealing. Indeed, while there is a well-documented reduction in liking for food tasted in the sated state (e.g. [8]), for any given food wanting declines *far more*. This ‘additional satiety'—the term we use here to refer to this effect—is robust and replicable (e.g. [9–12]). The main aim of this report is to investigate some of the psychological processes that may generate this ‘additional satiety'.

There is an obvious, but particularly important, distinction between the satiety evident for wanting and the satiety evident when tasting a food. Food wanting usually occurs in the absence of direct sensory stimulation of the oronasal chemosensory and tactile receptors that occur when a food is actually tasted (e.g. [13,14]). These chemosensory receptors are important for generating sensory liking, namely the pleasure from sweet, salty and umami tastes, and from the texture of fats (e.g. [15,16]). Wanting, on the other hand, relies on a different source of sensory input, this usually being the visual system (e.g. [17,18]), such as when people see attractive food in a store or at a restaurant buffet. If wanting occurs when a food is seen, the desire to eat is very likely to be based upon memories of the previous occasions when that food was eaten (e.g. [19,20]). And herein lies the important distinction—wanting depends upon recollected pleasure, while liking depends upon sensory-induced pleasure.

One approach that has informed thinking about why wanting may decrease more than liking after a meal (i.e. ‘additional satiety') is based on the idea of memory inhibition. This has been developed in animal models by Davidson and colleagues and is premised on the idea that food always serves as an excitatory cue [21,22]. However, during satiety, the excitatory effect of food cues is inhibited (e.g. [21,23,24]). This model has been well tested in animals and there is now a good understanding of its neural basis in the hippocampus, and evidence that satiety can serve as an inhibitory cue in the manner that the model proposes [25]. It has been suggested that the ‘additional satiety' that occurs for wanting may be a consequence of memory inhibition (e.g. [9,12]). Greater ‘additional satiety' has been linked to better hippocampal-dependent memory function (e.g. [9,12]), suggesting neural commonality with animal findings. However, the actual way that memory inhibition might operate in humans has received little attention, but at least two different accounts are plausible. It is these two accounts that we aim to test here.

In the first account, looking at or thinking about a palatable food involves retrieval of prior episodes of consumption that include some form of mental simulation/imagery of what that food is like to eat. This simulation involves the affective consequences of consumption (e.g. [26]). After eating a filling meal, and hence after a change in internal state, feelings of gastric fullness and the other interoceptive sensations that constitute satiety act to inhibit food memories entering consciousness. They do so because the interoceptive satiety state *itself* functions as a simulation of what it would feel like to eat *now* (another way of conceptualizing this is in terms of Wagner's [27] SOP model, with the interoceptive state (fullness) active in short-term memory and inhibiting food-induced retrieval). Consequently, during satiety, there is no need to access memory and engage in imagery/simulation, as the interoceptive satiety state is that simulation. The key prediction from this *interoceptive account* is that anything that affects retrieval of food-related memories and imagery/simulation, will reduce wanting when hungry, but have less impact when sated.

The second possibility is more aligned with the animal model of inhibition (e.g. [21,23,24]). In this account, as with the preceding one, wanting in the hungry state involves memory retrieval and imagery/simulation. However, after eating, satiety serves as an occasion setter [28], acting to inhibit retrieval of pleasant food-related memories, allowing more affectively negative memories of feeding to come to mind. These negative food memories then form the basis for simulations of what it might be like to eat now. Importantly, mental imagery and simulation of eating should be as engaged during satiety as during hunger, with just memory content differing between states—hence the *memory content account*. On this basis, any intervention that adversely affects retrieval of food memories, imagery/simulation, should exert an equal effect on wanting when hungry and sated.

**Figure 1.**
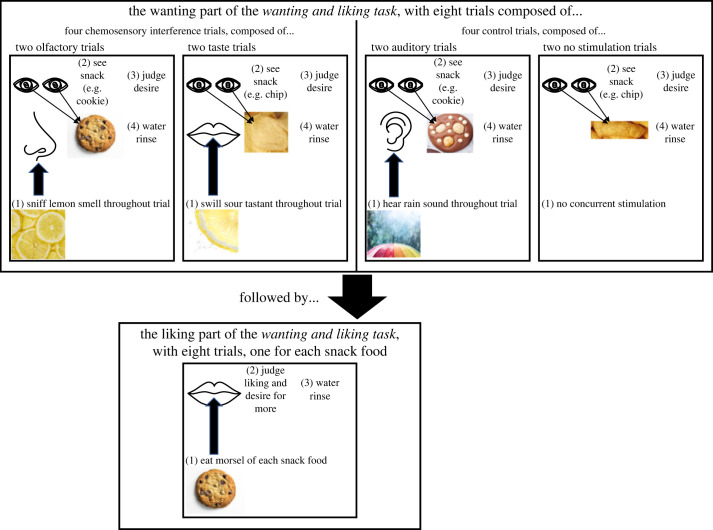
Diagrammatic summary of the procedure for the wanting and liking task.

To test these accounts, we used two approaches, both undertaken before and after a filling meal. The first employed the wanting and liking task, where participants look at palatable snack foods and rate their desire to eat them, followed shortly after by tasting each food and rating sensory liking. Here, we employed a method to suppress mental imagery/simulation of food-related content during some of the wanting trials (figure 1). This approach was based on the idea that mental imagery/simulation draws upon many of the same neurocognitive processes that are engaged during perception (e.g. [29–31]). Thus, attempting imagery/simulation in the same perceptual channel concurrently receiving sensory input should be disruptive (e.g. [32–34]). As the act of eating mainly involves taste, smell and texture (e.g. [13,14]), these were the senses that we targeted to impair food-related imagery/simulation. To this end, participants undertook four types of trials while they evaluated wanting. Two involved chemosensory stimulation—one using taste/texture and the other olfaction. The other two formed the control. One involved listening to a sound while evaluating wanting. Audition was selected as it is not normally involved in food imagery (e.g. [35]). The other just required wanting judgements with no concurrent sensory stimulation. The key question was whether chemosensory interference would reduce wanting equally across state (i.e. hungry = sated) or differentially (i.e. hungry > sated).

The second test employed the food recollection task, which involves deliberative memory retrieval of what it would be like to eat a particular food now (e.g. describe what it would be like to eat a steak). The memory content account suggests that food memories should become less affectively positive and more affectively negative across state (i.e. hungry to full). This is due to the inhibition of pleasant food-related memories during satiety, which allows negative memories to dominate. By contrast, the interoceptive account would suggest: (i) fewer affect-laden food memories entering consciousness in the sated state as the interoceptive state of fullness is ‘in mind'; (ii) those memories that are reported should include more frequent reference to how filling the food is, as the interoceptive state of fullness is ‘in mind' and should ‘contaminate’ recollections; and (iii) both (i) and (ii) should relate to how full the person feels (i.e. current interoceptive state). Finally, the main effects on memory predicted for the food recollection task (i.e. increase in negative and reduction in positive food-related memories across state versus reduction in the number of affect laden food memories across state) should correlate with the ‘additional satiety' effect on the wanting and liking task, providing a further test of the memory content and interoceptive accounts.

## 2. Method

### 2.1. Overview

The study used a wholly within-participant design. There were two main tasks, the food recollection task and the wanting and liking task. Both were undertaken before and after lunch. In the food recollection task, hungry participants wrote descriptions of what it would be like to eat two foods and touch one object. They then undertook the wanting and liking task. This involved two parts. First, judging desire for palatable snack foods while looking at them, either while concurrently receiving chemosensory interference (to impair chemosensory imagery) or control manipulations (figure 1). Second, eating a morsel of each snack food used in the first part, and rating it for liking (figure 1). Participants then ate a filling meal and repeated both tasks.

### 2.2. Participants

Forty-eight first year undergraduates (14 men) participated for either course credit or a small cash payment. Mean age was 19.6 years (s.d. = 2.8), with an average body mass index of 22.0 (s.d. = 3.0). The study was approved by the Macquarie University Human Research Ethics Committee and written consent was provided by each participant. The study was described as exploring the impact of hunger and fullness on ‘various psychological states'. The specific rationale for the study was explained at the end.

### 2.3. Stimuli

There were eight snack foods used in the wanting and liking task and these were organized into four pairs. Each pair was composed of one sweet and one savoury food. The four pairs were: one Cheetos cheese and bacon ball and one mini Oreo cookie; one Pringles salt and vinegar chip and one Tiny Teddy mini chocolate chip cookie; one Pringles BBQ chip and one Cadbury's mini chocolate finger; and one cube of cheddar cheese (0.5 cm^3^) and one Malteser. These snack food pairs were fully counterbalanced across the four types of trial used in the wanting part of the wanting and liking task (figure 1).

The concurrent stimuli used in the trials of the wanting part of the wanting and liking task (figure 1) were composed of an odorant, a tastant and a sound. The odorant was 25 mg of lemon oil on a cotton wool ball presented in an opaque 250 ml plastic squeezy bottle (i.e. a weak lemon odour). The tastant was 10 ml of 3.1 mM citric acid (i.e. a weak sour taste). The auditory stimulus was the sound of falling rain obtained from the international affective sound series and presented via a smartphone (i.e. a soft pattering of rain (50 dB)).

Lunch consisted of either ‘On the menu’ brand beef (260 g; 1570 kJ) or vegetable lasagne (260 g; 1140 kJ)—according to preference—and 100 g of vanilla ice-cream (Bulla; 718 kJ). Water was available ad libitum during lunch.

### 2.4. Procedure

A set of ‘general rating scales' were completed first. Participants rated their hunger, fullness, thirst, happiness, sadness, relaxedness and alertness, all using 120 mm visual analogue scales (anchors: ‘Not at all' and ‘Very'). Only hunger and fullness ratings are reported as the remainder served as distractors.

The first food recollection task followed. This started with a practice phase with the following instructions: ‘Please describe what it would be like to eat a meat pie and sauce right now. Please use as much detail (e.g. taste, flavour, smell, texture, attractiveness, etc.) as needed to describe what this experience would be like at this moment now’. Participants were told they would have 1 min and following this minute they were given a further 20 sec to complete their response. After this practice phase, participants were asked to provide descriptions of what it would be like to experience three objects—two foods (i.e. eating them) and one non-food (touching it). There were two sets of the three objects. This was so a different set could be used before and after lunch for each participant, with this being fully counterbalanced. One set was composed of: (i) a prime steak, salad and fries; (ii) a bowl of boiled rice and soy sauce; and (iii) a bean bag. The other set was composed of: (i) a rack of ribs, fries and onion rings; (ii) a bowl of noodles and soy sauce; and (iii) a suitcase. Objects within each set were presented in a fixed random order.

Participants then undertook the wanting and liking test. This had two parts (figure 1). The first involved eight trials. On some of these trials another stimulus—a smell, a taste or a sound—was experienced just *before*, and during the whole period in which they were both shown the snack food and rating their desire to eat it (How much would you like to eat this food? 120 mm VAS, anchors: Not at all, A lot). Only once this rating was complete did the concurrent stimulation cease. Importantly, the snack food was never eaten on any of these trials—it was just looked at. There were four trial types—olfaction, taste, audition and no concurrent stimulation—with each being undertaken twice, once for each food of the pair assigned to that imagery condition (i.e. one sweet and one savoury snack food). Before starting, participants undertook a practice trial for both the smelling and tasting procedures, using chocolate as the target food. The control and auditory tasks were just described to participants. The procedure for each trial type is illustrated diagrammatically in figure 1.

A taste trial involved pouring all of a 10 ml citric acid stimulus into the mouth and gently swilling it around. The target snack food was then brought into view, and the participant was asked to complete their wanting rating. Once this rating was completed, the food item was removed from sight and participants expectorated, rinsed with water, and then the second taste trial commenced. For an olfactory trial, the same basic procedure ensued, but this time participants continuously sniffed a lemon odorant (puffed at them by the experimenter) while they looked at and then rated the target snack food. This too was followed by a water rinse and then the second olfactory trial. For an auditory trial, the rain sound was looped so that it played across the whole period in which the participant looked at and rated the snack food. Again, this concluded with a water rinse and then the second auditory trial. For a no concurrent stimulation trial, participants just viewed and rated the target snack food, followed by both a water rinse and then their second no concurrent stimulation trial.

In the second component of the wanting and liking test, participants now ate a single morsel of each of the eight foods used in the first component (figure 1). Each trial was composed of eating all of the sample, rating how much they liked the food (120 mm VAS, anchors: Not at all, A lot) and how much more of it they would like to eat (120 mm VAS, anchors: None, A lot). This ‘want more' rating was included for continuity with our previous uses of the wanting and liking task and we did not expect any task-relevant outcomes for this measure. A water rinse again separated each trial. Food stimuli were presented in the same order as they were on the first component of this test, to ensure a similar judgemental context.

Following a second set of general scales (i.e. hunger, fullness, etc.), participants were served their lunch. They were encouraged to eat as much of the food as possible, so as to be comfortably full by the end of the meal. Prior to eating they were instructed: ‘This is the main meal. Please eat as much of this food as you can. All the food that is uneaten will be thrown away. Please feel free to read the magazines while you eat—if you like. I will step outside while you eat'. Participants were then left for 5 min, with the experimenter then returning to remove any uneaten lasagne for later weighing. The ice-cream was then presented in the same manner (i.e. with 5 min for eating), with uneaten ice-cream weighed at the end of the meal. Participant then completed a further set of general rating scales.

After lunch, participants completed the second wanting and liking task (this being separated from the first task by about 20 min), with this being undertaken in an identical manner to the first. The second food recollection task followed, with presentation as described above, but using the set of stimuli not employed in the pre-lunch test. Biographical data were then obtained, alongside frequency of consumption of the test foods used in the wanting and liking task, and ratings of liking for the concurrent stimuli used in the wanting part of the wanting and liking task (120 mm VAS, anchors: dislike, indifferent, like). Participants were also asked to rate how well they could form visual, flavour and textural imagery (120 mm VAS, anchors: Not at all, Good). Participants were then weighed and had their height measured and were provided with a verbal debriefing of the study.

## 3. Analysis

Only the food recollection data are reported (the non-food recollection data were included to avoid a sole focus on food). These data were coded for the presence of positive affect (a score of 1 for each mention), negative affect (a score of 1 for each mention), and for any report of how filling it would be to eat (with a score of 1 for each mention). The primary coder coded every response for every participant. The reliability coder recoded 12 randomly selected participants' data from the full set of 48 cases. Both coders were blind to participant state and to the study aims. To assess reliability, scores were first collapsed both across foods and state, so as to increase variability. Reliability was then examined using the intra-class correlation coefficient (ICC), between the primary and reliability coder. The ICC for food positive hedonics was = 0.93, for food negative hedonics = 0.83 and for food fillingness = 0.89, thus indicating excellent to good reliability.

On the wanting and liking task, we conducted preliminary analyses on the wanting data to check if there were any differences between the two types of chemosensory trial (taste versus smell), between the two types of control trial (sound versus no concurrent stimulation) and then between the chemosensory trials and the sound trials. As planned, and also because there were no significant differences, we combined the taste and smell trials to form the chemosensory interference condition, and the sound and no concurrent stimulation trials to form the control condition.

All data were suitable for parametric testing, excepting that from the food recollection task, where non-parametric tests were employed.

## 4. Results

### 4.1. Manipulation of state

Participants consumed a mean of 221 g of lasagne (s.d. = 31 g) and 79 g of ice-cream (s.d. = 11 g). Hunger and fullness ratings were obtained at the start of the experiment, and before and after lunch. These ratings were analysed using a two-way repeated measures ANOVA with Time and Rating type as factors. There were main effects of Time (*p* < 0.001) and Rating type (*p* < 0.01), and an interaction between these variables (*F*_2,94_ = 199.15, m.s.e. = 657.24, partial *η*^2^ = 0.81). Hunger ratings changed little (M = 79/120 to M = 77/120) prior to lunch, and fell markedly after lunch (M = 22/120). Fullness ratings increased prior to lunch (M = 15/120 to M = 32/120), and increased more after lunch (M = 94/120). The manipulation of state was then successful.

### 4.2. Wanting and liking task

We started by analysing the wanting, liking and want more ratings individually, using in each case a two-way repeated measures ANOVA with State (hungry versus full) and Interference type (chemosensory versus control) as factors. The results for each ANOVA are presented in table 1, and the data are presented in table 2. As expected, there was a substantial effect of State for each rating type, with all responses declining when sated.

**Table 1.** Analysis of the wanting, liking and want more ratings.

rating	ANOVA outcome			
effect	*F*_1,47_	*p*	m.s.e.	*η*^2^
wanting				
State	76.82	0.001	425.01	0.62
Interference type	11.32	0.002	314.91	0.19
interaction	2.67	0.109	86.13	0.05
liking				
State	84.66	0.001	177.79	0.64
Interference type	0.11	0.747	269.91	0.00
interaction	4.01	0.051	35.51	0.08
want more				
State	122.46	0.001	368.93	0.72
Interference type	0.03	0.856	222.99	0.00
interaction	0.05	0.829	88.26	0.00

**Table 2.** Mean wanting, liking and want more ratings across state and interference type.

rating	Interference type	
State	chemosensory M (s.d.)	control M (s.d.)
wanting		
hungry	64.3 (24.3)	75.1 (21.0)
sated	40.4 (23.7)	46.8 (25.6)
liking		
hungry	82.7 (17.9)	83.6 (16.1)
sated	66.7 (21.2)	64.2 (20.1)
want more		
hungry	68.0 (24.3)	68.1 (25.2)
sated	37.1 (26.0)	37.7 (25.7)

For wanting ratings, there was a main effect of Interference type. *Post hoc* tests revealed that chemosensory interference significantly reduced wanting ratings both in the hungry (*t*_47_ = 3.28, *p* = 0.002) and sated (*t*_47_ = 2.65, *p* = 0.011) states. There was no evidence that this interference effect differed by state (i.e. non-significant interaction). This pattern of findings is most consistent with the idea that imagery/simulation occurs equally in both the hungry and sated states—that is the memory content account.

For liking ratings, we had no expectation that the interference manipulation would affect responding because no interference manipulation was undertaken while these ratings were made. Consistent with this, there was no main effect of Interference type. However, and unexpectedly, the interaction of State and Interference type approached significance, with a smaller change in liking between the hungry and stated states in the chemosensory interference condition. Finally, want more ratings revealed no effect of Interference type.

**Figure 2.**
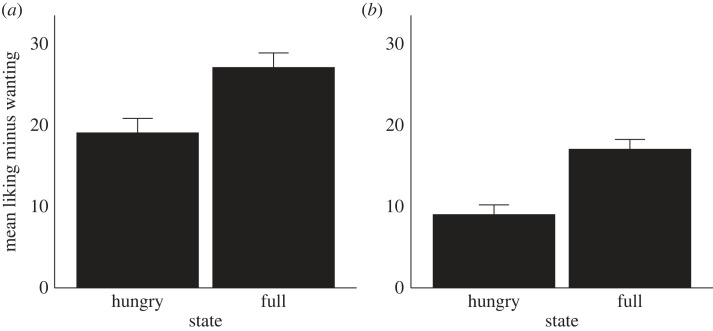
The ‘additional satiety' effect (greater reduction in liking minus wanting across state) for the chemosensory (*a*) and control condition (*b*), with standard error.

To test for the ‘additional satiety' effect, and to check if it differed between the chemosensory interference and control condition, we conducted two sets of contrasts. First, we tested if the change in wanting across state exceeded the change in liking in the chemosensory interference condition, and then in the control condition (figure 2). There was a significant ‘additional satiety' effect for both the chemosensory interference condition (*t*_47_ = 2.46, *p* = 0.018) and the control condition (*t*_47_ = 2.77, *p* = 0.008). In other words, and as evident in figure 2, wanting declined more across state (i.e. ‘additional satiety') relative to liking. Second, we examined if these two effects differed, but they did not (*p* = 0.77).

### 4.3. Food recollection task

Food recollections were coded for inclusion of hedonic terms (positive, negative and their total) and for any mention of how filling the food would be to eat (table 3). When sated, food recollections contained significantly fewer positive hedonic comments and significantly more negative hedonic comments, than when hungry—consistent with the memory content account. However, while there was no reduction in the total number of affect-laden reports between states, reports of how filling the food was were significantly increased from the hungry to the sated state, providing some support for the interoceptive account.

**Table 3.** Data from the food and non-food recollections task.

coded response	M responses per person (s.d.)		comparison using a
	hungry	sated	Wilcoxon test
positive hedonic	2.6 (1.8)	1.9 (1.9)	*Z* = −2.74, *p* = 0.006
negative hedonic	1.4 (1.6)	2.1 (1.8)	*Z* = 2.44, *p* = 0.015
total affective responses	4.0 (2.3)	4.1 (2.3)	*Z* = 0.22, *p* = 0.83
fillingness	0.4 (0.8)	0.6 (1.0)	*Z* = 2.13, *p* = 0.033

We then tested the relationships identified in the Introduction. First, we examined two predictions pertinent to the interoceptive account. There was a significant association between changes in fullness across the study and changes in total affective responses (*ρ* = 0.33, *p* = 0.024), but this was *not* in the expected direction. That is more affectively laden instances of memory during satiety (relative to when hungry) were described by those with the largest changes in fullness. There was no association between changes in fillingness and changes in fullness (*ρ* = 0.06, *p* = 0.71).

We then examined relationships between the food recollection task and the wanting and liking task. There was a significant relationship between changes in the effect of retrieved food memories on the food description task (i.e. less affectively positive and more affectively negative across state) with the ‘additional satiety' effect (i.e. the greater decline in wanting relative to liking) from the wanting and liking task (*ρ* = 0.32, *p* = 0.027) providing support for the memory content account. There was no significant relationship between changes in total affective responses on the food description task and the ‘additional satiety' effect from the wanting and liking task (*ρ* = −0.02, *p* = 0.92), contrary to expectations for the interoceptive account.

### 4.4. Other variables

We examined participants' hedonic evaluations of the concurrent task stimuli used in the wanting phase of the wanting and liking task. There was no significant difference between the auditory (M = 70/120) and olfactory (M = 64/120) stimuli (*t* < 1), but the tastant (M = 48/120) was judged as less pleasant than these other two (*p*s < 0.014). Participants were also asked about their capacity for mental imagery. Visual imagery was reportedly better (M = 89/120) than both textural (M = 78/120; *p* = 0.011) and flavour (M = 73/120; *p* = 0.001) imagery, which did not differ (*t* = 1.3). Finally, participants reported moderate consumption of each of the snacks used in the wanting and liking test (monthly to weekly).

## 5. Discussion

Participants were asked to judge both their desire (wanting) for palatable snack foods, and how much they liked their taste, before and after a filling lunch. Half of their wanting ratings were undertaken with concurrent chemosensory interference, the remaining half without. Chemosensory interference reduced participants' desire (wanting) for palatable snack foods. The key test was to see if this reduction was of an equivalent magnitude when they were hungry and when they were sated. The analysis revealed that chemosensory interference reduced desire by a similar degree across state. We then contrasted wanting and liking ratings, finding that the ‘additional satiety' effect was present in both the chemosensory interference and control conditions, and to a similar degree. Participants were also asked to recollect what it would be like to consume particular foods, undertaking this description task before and after lunch. When participants were sated, their recollective reports contained more negative and fewer positive comments about the foods, and more statements about the food's fillingness, relative to when they were hungry. The change in positive/negative recollections across state on the food recollection task was significantly correlated with the ‘additional satiety' effect from the wanting and liking task.

The primary aim of this experiment was to test two accounts of how memory inhibition might result in the observed ‘additional satiety' effect, where desire for palatable snack foods when full, is far less than the actual pleasure reported when they are tasted. Both accounts draw upon the same mechanism when hungry participants see palatable snacks. Namely, memories of these foods are retrieved and used to construct mental images enabling simulations of eating. However, when sated, the interoceptive account suggests that fullness sensations serve as a simulation of what eating will be like now, negating the need to retrieve food memory and engage imagery. Crucially, for this account, food memory retrieval and imagery are necessary for the hungry state but not for the sated state. By contrast, for the memory content account, satiety cues act to inhibit retrieval of pleasant food-related memories, resulting in retrieval of affectively negative memories. Thus, the hypothesized process during satiety requires mental imagery and simulation, in the same way as it does when hungry. The basic findings from the current experiment are most consistent with the memory content account. This is because, in the analysis of the wanting data, the chemosensory interference manipulation reduced desire significantly and equally in both the hungry *and* sated state, consistent with the operation of food memory retrieval and mental imagery/simulation occurring in both states.

An important consideration in respect to this conclusion concerns the nature of the interference manipulation in the wanting part of the wanting and liking task (figure 1). There were two basic assumptions in its design. First, that in retrieving memories of these foods and then using these to form mental images or simulations of eating them, participants would use chemosensory perceptual systems, namely drawing upon taste and smell (e.g. [35]). Second, irrespective of how conscious and explicit these simulations were, data suggest that many of the same brain areas used during perception are activated during mental imagery and simulation (e.g. [29–31]). We reasoned on this basis, as have others before (e.g. [32–34]), that asking participants to undertake a concurrent task that draws upon a sensory modality likely to be involved in mental imagery/simulation would disrupt it. By contrast, concurrent tasks using sensory systems not used in imagery/simulation should have little effect. Consistent with this, we found no evidence that listening to rain impacted wanting ratings, while both chemosensory tasks—taste/texture and smell—did. The similarity of the two chemosensory conditions, and their difference from the auditory condition are important for a further reason. Participants rated their liking for the concurrent stimuli, with no difference found for smell and sound (both mildly positive), while taste was judged as significantly less pleasant. That these hedonic differences do not align with the impacts on wanting ratings suggests that the central aspect of our interference manipulation was its use of a particular sensory modality, rather than reflecting differences in affect.

The study also included a food recollection task, where participants were asked to describe what it would be like to eat certain foods and to touch certain objects. Participants made more positive and fewer negative comments in their food recollections when they were hungry, than when sated. This change is also consistent with the memory content account. We suggested in the Introduction that recollective differences between the two states, especially in regard to affect, are a key feature of the memory content account. This conclusion is given further support by the finding that changes across state in the effect of participants' food recollections on the food description task correlated with the ‘additional satiety' effect. This implies that a common mechanism is responsible for both effects, and we suggest that this is memory inhibition generated by satiety cues and operating on the retrieval of food memories (e.g. [21,23,24]).

As we outlined above, the interoceptive account was also tested here. There were two findings from the current study that suggest it would be premature to dismiss this account. The first concerns the wanting ratings. The interaction of Interference type and State (table 1) had medium effect size—and if tested with a directional paired-sample *t*-test, alpha would have been 0.0545. The point here is that even though the chemosensory interference task unequivocally reduced desire both when participants were hungry and sated, these data still suggest some modest reduction under conditions of satiety. The second relevant finding comes from the Food recollection task, where participants reported more comments pertaining to the filling nature of the food when they were sated than when they were hungry. This effect has been obtained before when using this type of task [36]. In this prior report, we also found that measures of gastric sensation—stomach bloating, stomach emptiness and nausea (but not fullness)—were correlated with changes in reports of fillingness, between the hungry and sated state. Indeed, it was these findings which led us to suggest that one way that memory inhibition might operate is for the interoceptive cues associated with fullness to dwell in consciousness to the extent that they negate the need to simulate eating a food. This is arguably because the interoceptive state effectively functions as a simulation of what eating will feel like *now*. There is of course no reason why the two accounts need to be mutually exclusive, and it may be that the type of interoceptive model suggested by reports of fullness is operative, but is dependent upon the extent to which stomach-related sensations are evident after a meal. This in turn would depend both on individual differences in interoceptive sensitivity (e.g. [37]) and on the quantity of food consumed, such that the stronger these stomach-related signals become, the more unnecessary any eating-related simulation would be.

For liking ratings, there was some—albeit non-significant (*p* = 0.051)—indications that the foods used in the chemosensory interference condition changed less in liking across state than those used in the control condition (see data in table 2). This was unexpected, because the interference manipulation was not undertaken while participants made liking judgements (figure 1). As to why this may have occurred, one possibility is that the lower levels of desire for the chemosensory condition foods generated by the chemosensory interference condition may have created a positive contrast effect when these foods were actually tasted. This may not have been so apparent when participants were hungry, perhaps because of ceiling effects for the liking ratings.

Finally, it is worth reflecting on the broader implications of these findings. Currently, we do not know if the ‘additional satiety' effect is operative for other motivational systems such as thirst, sex and drugs. If it is, and this would seem plausible, then this would have some interesting implications. The principal one comes from the finding that memory inhibition—however it operates—is very sensitive to subtle hippocampal impairment even in otherwise healthy people [9,12]. As impairments in memory inhibition—that is a reduction or loss of ‘additional satiety'—can be induced by one week of a diet rich in saturated fat and added sugar, in people who normally eat a fairly healthy diet, the suspicion would emerge that thirst, sex and drug use might similarly be impacted. This would have the effect of making pleasant tasting beverages, attractive mates and previously used narcotics more appealing than they otherwise would be during satiety.

In conclusion, the experiment reported here examined two possible models of how memory inhibition may operate to drive the ‘additional satiety' effect. The findings are most consistent with the memory content account in which memory retrieval, imagery and simulation of eating occur both when hungry *and* when sated participants view food, with the difference between the two states being the nature of the recollected material—memory content. This type of recollective difference was illustrated in the food recollection task, with participants reporting more positive and fewer negative hedonic comments in their food descriptions when hungry relative to when sated. Finally, the affective changes observed on the food recollection task were found to correlate with the changes in wanting relative to liking (i.e. ‘additional satiety') on the wanting and liking task.

## Data Availability

The data are provided in the electronic supplementary material [38].
